# Wnt-signalling pathways and microRNAs network in carcinogenesis: experimental and bioinformatics approaches

**DOI:** 10.1186/s12943-016-0541-3

**Published:** 2016-09-02

**Authors:** Emenike K. Onyido, Eloise Sweeney, Abdolrahman Shams Nateri

**Affiliations:** Cancer Genetics & Stem Cell Group, Cancer Biology Unit, Division of Cancer & Stem Cells, School of Medicine, University of Nottingham, Nottingham, NG7 2UH UK

**Keywords:** Cancer, Cancer stem cell, miRNA, Wnt-signalling, Next generation sequencing, sRNA-Seq and ChIP-Seq

## Abstract

Over the past few years, microRNAs (miRNAs) have not only emerged as integral regulators of gene expression at the post-transcriptional level but also respond to signalling molecules to affect cell function(s). miRNAs crosstalk with a variety of the key cellular signalling networks such as Wnt, transforming growth factor-β and Notch, control stem cell activity in maintaining tissue homeostasis, while if dysregulated contributes to the initiation and progression of cancer. Herein, we overview the molecular mechanism(s) underlying the crosstalk between Wnt-signalling components (canonical and non-canonical) and miRNAs, as well as changes in the miRNA/Wnt-signalling components observed in the different forms of cancer. Furthermore, the fundamental understanding of miRNA-mediated regulation of Wnt-signalling pathway and vice versa has been significantly improved by high-throughput genomics and bioinformatics technologies. Whilst, these approaches have identified a number of specific miRNA(s) that function as oncogenes or tumour suppressors, additional analyses will be necessary to fully unravel the links among conserved cellular signalling pathways and miRNAs and their potential associated components in cancer, thereby creating therapeutic avenues against tumours. Hence, we also discuss the current challenges associated with Wnt-signalling/miRNAs complex and the analysis using the biomedical experimental and bioinformatics approaches.

## Background

The Wnt pathway is a highly regulated signalling pathway that controls numerous stages of animal development and tissue homeostasis. The Wnt proteins comprise a highly conserved and diverse family of genes found in humans, mice, Xenopus, Zebrafish and Drosophila [1] The pathway is closely regulated at both transcriptional-level regulations to post-translational modification; thus aberrant Wnt activity often results in developmental disorders and diseases including but not limited to cancer [2–4]. For example, during metastatic processes, epithelial cancer cells require certain characteristics such as elevated expression of mesenchymal markers as well as other alterations in their microenvironment to enable invasion of adjacent tissues and progression to metastatic high-grade tumours. Inappropriate Wnt signals coupled with a loss of E-cadherin promotes an increase in cytoplasmic and nuclear β-catenin levels where it interacts with the epithelial-mesenchymal transition (EMT) regulators, such as E-cadherin repressors: Snail, twists and Zebs [5, 6]. MicroRNAs (miRNAs) are composed of ~22 nucleotide sequences in length and belongs to the class of non-coding endogenous small RNAs that are integral post-transcriptional regulators of the gene expression via direct interaction with the 3’un-translated region (UTR) of the target messenger RNAs [7]. Recent advances in biomedical research have allowed experimental and bioinformatics approaches to identify short non-coding RNAs such as microRNAs (miRNAs) as regulators of components of the Wnt-signalling pathways and vice versa. Thus, both miRNAs and Wnt-signalling pathways form a network involved in the regulation of key biological processes.

## Main text

### Canonical Wnt-signalling

**Fig. 1:**
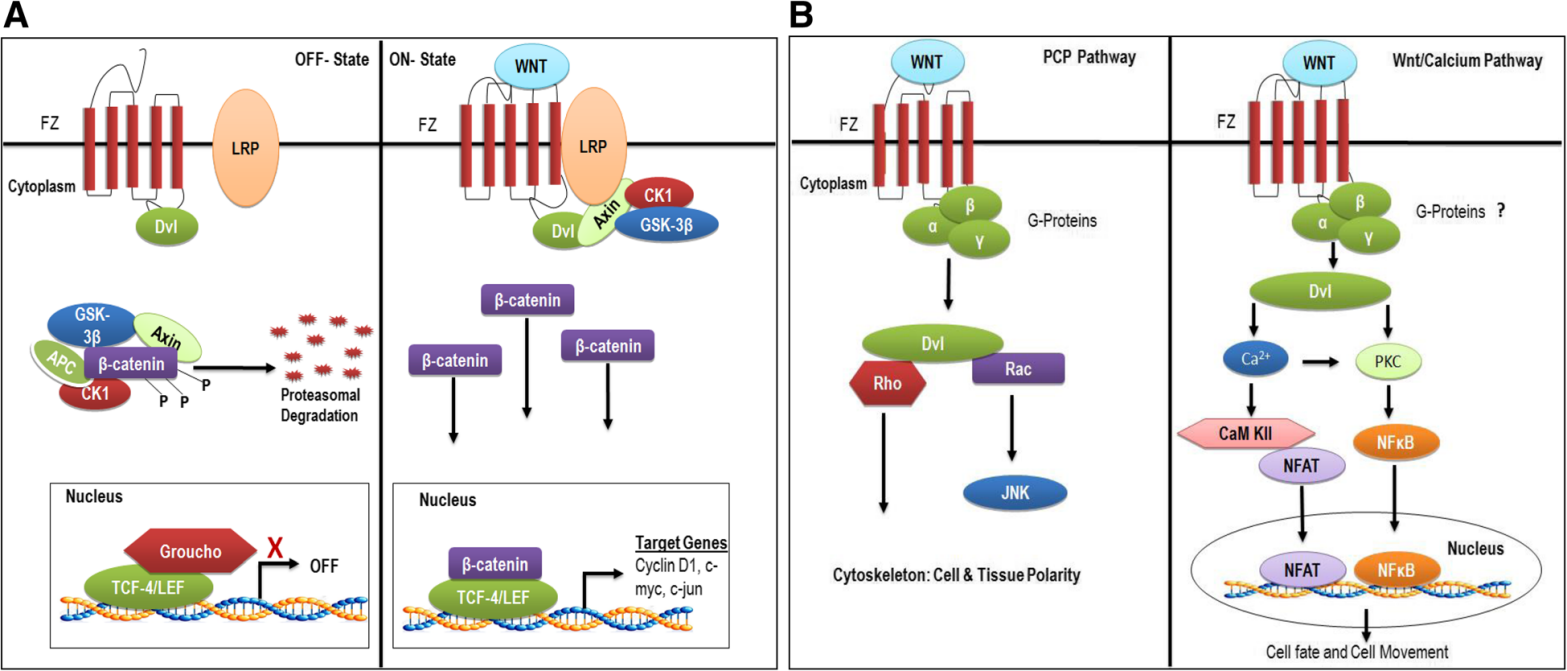
**a** Representation of Canonical Wnt/β-catenin pathway. *OFF- State:* β-catenin is regulated by the destruction complex in the absence of Wnt ligands. GSK-3β and CK1 facilitates the phosphorylation of β-catenin at specific serine and threonine sites rendering it a target for proteosomal degradation by β-TRCP. As a result of this degradation, β-catenin is prevented from translocating into the nucleus prompting Groucho (co-repressor) to be bound to TCF thereby repressing gene transcription. *ON- State:* Once binding of Wnt ligand to Fzd and LRP5/6 co-receptors occurs, Dvl-fzd complex is formed resulting to the phosphorylation of LRP5/6 by GSK-3β and triggering the recruitment of Axin2 from the destruction complex. The disassembly of the complex promotes stabilization and accumulation of cytoplasmic β-catenin which eventually translocate to the nucleus where Groucho is dislodged and TCF is converted into a transcription factor ensuring the transcription of many genes including *c-Myc, Ascl2, cyclin D1* which are essential stem cell regulators as well as mediators of proliferation and differentiation. **b** Schematic of Non-Canonical Wnt pathway. In the PCP pathway, Fz activates Dvl through G-proteins in the absence of LRP receptors. Subsequent activation of the Rho GTPases, Rho and Rac results to the induction of cytoskeletal changes. In the Wnt/Calcium pathway, Dvl activates protein kinase C (PKC) and the release of intracellular calcium and calcium/calmodulin-dependent protein kinase II (CaMKII) which in turn activates the release of NFAT and NFkB. NFAT and NFkB subsequently translocate into the nucleus to transcribe regulatory genes that govern cell migration. It is still unclear whether G-proteins are involved in this particular pathway. Adapted from [209]

The canonical Wnt-signalling cascade refers to the transduction of series of signals mediated via the interaction of specific Wnt ligands with their target receptor resulting in the accumulation of β-catenin (Fig. 1a). Amassment of β-catenin plays a crucial role as the central transducer in the activation of downstream factors [8]. The cytoplasmic stability of β-catenin is usually maintained at a minimal level by the destruction complex composed of a scaffold combination of tumour suppressor protein adenomatous polyposis coli (APC), Axin2, casein kinase1 (CK1) and glycogen synthase kinase 3β (GSK-3β) [9]. Aberrant Wnt/β-catenin signalling is a common hallmark of malignant CRC cells hence, mutations in any of the components of the destruction complex can potentially result to cytosolic β-catenin accumulation and subsequent activation of Wnt target genes that drive proliferation [10, 11].

In the absence of Wnt ligand interaction (*OFF-state*), the membrane receptor complex is inactivated, thereby preventing the clustering of a trimeric complex composed of Wnt ligand/Fzd/LRP receptors and eventually results to β-catenin ubiquitination and degradation (Fig. 1a) [11]. Although the Wnt/Fzd/LRP complex model is widely accepted, the precise mechanism of formation is yet to be fully understood. The current and widely accepted model suggests that CK1 and GSK-3β kinases target β-catenin by phosphorylating a set of conserved threonine and serine residues located in the amino acid terminus [10, 12]. This phosphorylation occurs simultaneously at specific sites, with Serine 45 (Ser45) N-terminus of β-catenin phosphorylated by CK1 while phosphorylation at Threonine 41 (Thr41), Ser33 and Ser37 sites is carried out by GSK-3β [8, 13]. The consequence of these series of phosphorylation activities results in the recruitment of APC to the destruction complex. The APC protein forms a synergy with other components of the destruction complex and mediates the degradation of cytoplasmic β-catenin. APC serves a very important role in the destruction complex due to its tumour suppressor properties [9, 14], as numerous scientific evidences show mutations in the *APC* gene are not only responsible for familial adenomatous polyposis (FAP), but also plays a significant rate-limiting role in the initial stages of majority of sporadic colorectal cancers [14, 15]. Subsequently, the phosphorylated regions of β-catenin are exposed to the F-box/WD repeat protein which is a component of E3 ubiquitin ligase complex β-transducin repeat-containing protein (β-TRCP) which mediates the ubiquitination and the proteasomal degradation of β-catenin [1]. Once β-catenin is degraded Wnt target genes fails to be transcribed. This process keeps the cytoplasmic β-catenin level low thereby preventing the translocation of β-catenin into the nucleus.

Conversely, the *ON-state* of canonical Wnt-signalling is activated by the binding of Wnt ligands, (secreted mainly by myofibroblasts beneath the base of the crypts in the small intestine), to Frizzled and LRP 5/6 co-receptors at the cell surface [16, 17]. Additionally, studies show that cancer associated fibroblasts (CAF) is associated with the canonical Wnt-signalling pathway [18], with stromal fibroblasts also implicated in the secretion of pro-tumourigenic factors that promote skin squamous cell carcinomas [19]. Formation of this receptor complex triggers the activation of Dsh/Dvl, a cytoplasmic scaffolding protein crucial for Wnt-induced LRP6 phosphorylation [20, 21], which proceeds to inhibit GSK-3β enzyme activity, triggering a complex series of events that prevents the phosphorylation and subsequent degradation of β-catenin resulting to its consequent stabilization and accumulation in the cytoplasm. Accumulated cytoplasmic β-catenin then translocate to the nucleus, where it displaces Groucho (a co-repressor transcriptional factor) due to its stronger affinity to engage TCF/LEF transcription factors [22, 23] to activate transcription of target Wnt genes such as c-*myc*, *c-jun*, *Axin2*, *Eph*B/*ephrin*-B and *Cyclin*D1. These target genes all play crucial roles in the regulation of a plethora of cellular processes including but not restricted to cell proliferation, cell division, stem cell maintenance as well as regulation of various stages of embryogenesis [24], hence a major consequence of dysregulation of β-catenin could result to maintenance of cancer phenotype [16, 25–27]. Interestingly, the progression of tumours to invasive cancers and metastatic disease may also involve the switch from canonical to non-canonical Wnt-signalling [28–31].

### Non-canonical Wnt-signalling

Apart from the canonical Wnt-pathway, specific Wnt-ligands can also trigger non-canonical pathways which are also referred to as β-catenin independent pathways (Fig. 1b). As earlier mentioned, the Wnt ligands consists of a large family of 19 secreted glycoproteins that are cysteine-rich and highly hydrophobic. It is not entirely understood which particular Wnt ligand participates in either of the Wnt-pathways, however, some Wnts (such as Wnt5a and Wnt11) initiate the β-catenin-independent pathways in a cell-specific independent manner [32]. The non-canonical Wnt pathways are further divided into three other distinct branches namely, the Wnt/Ca^2+^, the Wnt/JNK and the Wnt/planar cell polarity (PCP) pathways (Fig. 1b). The mechanisms of downstream signal transduction through these pathways is less understood, although scientific evidences suggest the 3 branches of the non-canonical pathway function dependently of each other [13].

Interestingly, the non-canonical Wnt-signalling has been reported to antagonize the β-catenin-dependent Wnt-signalling pathway by either or both CaMKII pathway and NFAT-mediated transcriptional regulation [33], however, other studies suggest that the PCP pathways can also antagonize the canonical Wnt cascade [34, 35]. The CaMKII pathway suppresses β-catenin-TCF-dependent transcription through phosphorylation of TCF transcription factors [36], while NFAT is reported to inhibit β-catenin-dependent transcription [37]. All these findings simply highlight the possible crosstalk among various types of Wnt-signalling, in addition to other multiple cell signalling cascades.

Recent advances to date has strongly cemented the fact that Wnt-signalling plays crucial roles in normal biological functions [26, 38]. Supporting scientific evidences over the past decade have successfully identified various signalling components that have contributed immensely to the establishment of a molecular framework for the understanding of the different branches of the Wnt signal transduction pathway, thereby enabling the correlation and causative relationships between human diseases and aberrant Wnt-signalling [38–40].

### Deregulation of Wnt-signalling activity via genetic mutations in cancer cells

Generally, cancerous genetic mutations on any gene that participates in the Wnt/β-catenin signalling cascade inappropriately reactivate the pathway, leading to abnormal ON-state activity. In effect, the activity of Wnt-signalling in the cell is no longer governed by the presence of Wnt ligands. Therefore, Wnt-signalling may be switched into the ON-state even in the absence of Wnt ligand–receptor interaction, leading to the characteristic sequence of ON-state activity: disassembly of the destruction complex, loss of phosphorylation and degradation of β-catenin and activation of Wnt-induced gene transcription, which all contributes to cancer progression. Mutations in *CTNNB1*, the gene for β-catenin, have been implicated in colon cancer, gastric cancer, medulloblastoma, melanoma, ovarian cancer, pancreatic cancer, and prostate cancer [41, 42]. Mutations in *APC* gene have frequently been identified in colon cancer as well, while mutations in *AXIN1*, the gene for Axin1, have been identified in hepatocellular carcinoma [43] and medulloblastoma [44]. Thus, there is considerable evidence for abnormalities in the Wnt-signalling pathway in tumorigenesis.

A major consequence of mutations in *APC*, *Axin*, *GSK-3*β and β*-catenin* genes is the increased nuclear β-catenin levels which has already been proven as a hallmark of intestinal tumorigenesis. Consequently, mutant APC protein becomes unable to form a proper destruction complex with GSK-3β which inevitably impairs the phosphorylation of β-catenin on specific sites. Similarly, mutations resulting to amino acid substitutions in the phosphorylated residues of β-catenin may hinder phosphorylation and recognition by the E3 ligase ubiquitin system thereby prompting the cytoplasmic stabilization and nuclear translocation of β-catenin [45]. Either form of disruption eventually promotes nuclear β-catenin accumulation which ultimately causes abnormal propagation of Wnt-signalling pathway. In addition, mutation in β-catenin gene *CTNNB1* can ultimately instigate a gain of function mutation which activates β-catenin, increasing its transcriptional activity and enabling the protein to evade phosphorylation and subsequent degradation [46, 47]. Furthermore, mutations also occur in Frizzled genes resulting in loss of the functional Wnt-receptor interaction responsible for the initiation of the Wnt-signalling cascade. These genetic mutations ultimately prevent the initiation of both canonical and non-canonical signalling cascade, thus compromising the integrity of the intestinal epithelium particularly the crypt base region [48, 49].

### Overview of miRNAs biogenesis

MicroRNAs (miRNAs) are composed of ~22 nucleotide sequences in length and belong to the class of non-coding endogenous small RNAs. The functions of miRNAs in posttranslational gene regulation involves the direct interaction of miRNA with the 3’ un-translated region (UTR) of the target messenger RNAs [7, 50]. In mammals, impartial base pairing between the miRNA and the target mRNA or gene also results in translational repression of the target gene [51]. To date, more than 2500 miRNAs have been identified in the human genome [52, 53]. The miRNA biogenesis is primarily mediated by the microprocessor complex composed of two RNase III endonucleases; Drosha and DGCR8 [52, 54]. In the first step of miRNA biogenesis, miRNAs are initially transcribed by RNA polymerase II resulting in the production of primary miRNA (pri-miRNA). Shortly after transcription and prior to translocation from the nucleus into the cytoplasm, the pri-miRNA undergoes modifications and transformation to a ~60–100 nucleotide hairpin structure precursor miRNA (pre-miRNA) via the activity of Drosha. Once the pre-miRNA is transported to the cytoplasm by Exportin-5 [7], it undergoes further modifications by Dicer, a multi-domain ribonuclease III enzyme. The cleavage event carried out by Dicer results to final product consisting of a 22 nucleotide double-stranded miRNA composed of a mature miRNA strand and its complementary miRNA strand [55].

Additionally, apart from the canonical miRNA biogenesis pathways described above, alternative mechanisms, particularly through mRNA splicing [56], have been discovered to generate miRNAs or other non-coding RNAs [57, 58]. The revelation of unconventional miRNA biogenesis has been made possible via deep sequencing of small RNAs from cells deficient in Drosha, Dgcr8 or Dicer which can be produced in a Dicer-independent or microprocessor-independent manner.

### Alterations of miRNAs/Wnt/β-catenin signalling in cancer

Numerous scientific evidences have implicated miRNAs as regulators of the Wnt-signalling pathway in the context of embryonic [59], osteoblast differentiation and bone formation [60, 61] and cardiac development [62, 63]. Bone metastasis occurs due to migration of cancer cells from the primary tumours cite to the bone causing bone alterations such as osteolysis and bone fracture [64]. Studies have implicated lung, prostate and breast cancer as the most frequent site of origin of metastatic cancer accumulation in bone [65, 66]. CXCR4, which is expressed in malignant breast tumours and human breast cancer cells have been shown to play a major role in site-specific metastasis of breast cancers to the bone. Interestingly, CXCR4 ligand CXCL12/SDF-1α is prominent in bone marrow stromal cells with studies showing that CXCR4 cooperatively with other factors such as IL-11, CTGF and OPN facilitate osteolytic bone metastasis in breast cancer MDA-MB-231 cells [67, 68]. Epithelial-mesenchymal transition (EMT) is a pathological event largely associated with tumour metastasis with studies demonstrating Wnt-signalling through Snail1 and Zeb1 regulates bone metastasis in lung cancer [65]. Additionally, miRNAs such as miR410 have been implicated in promoting growth, migration and invasion of NSCLC cells by activating the canonical Wnt-signalling *in-vitro* [69]. The network involving both miRNAs and Wnt-signalling pathway have been implicated in regulating tumorigeneses in brain cancer [70], colorectal cancer [59, 71], breast cancer [72], liver cancer [73] and other forms of cancer [74–78]. For instance, studies carried out on miR-374a via the use of immunofluorescence staining technique and subcellular fractionation showed that miR-374a overexpression resulted in the stabilization and accumulation of nuclear β-catenin in 4 T1 and MCF7 breast cancer cell lines [79]. This result suggests that miR-374a may be responsible for the degradation of APC or one of the other components of the destruction complex, hence leading to the translocation of β-catenin to the nucleus thereby enhancing the transcriptional activity of LEF/TCF4 [80].

Another miRNA, miR-200a, has been identified as a potential negative regulator of the Wnt/β-catenin signalling pathway. This miRNA targets mRNAs of Zeb1 and Zeb2 which are repressors of E-cadherin. Therefore, by degrading Zeb1 and Zeb2 mRNA, E-cadherin becomes abundant and available for binding with β-catenin thereby forming E-cadherin-β-catenin complex which promotes cell-cell adhesion [77]. The recruitment of β-catenin in the formation of this complex is beneficial towards reducing cytoplasmic β-catenin amassment which would eventually translocate to the nucleus and trigger the transcription of Wnt target genes [81]. Additionally, activation of Lin28 has been shown to be necessary for Wnt-β-catenin pathway mediated let-7 repression and cell proliferation [82]. Interestingly, let-7 miRNAs have also been implicated as a potential regulator β-catenin in cancer cells as overexpression of let-7a in Wnt activated MDA-MB-231 cells was observed to inhibit β-catenin-activated cell growth and colony formation, thus emphasising the significance of let-7 miRNAs as downstream regulators of Wnt-β-catenin pathway in the regulation of cell proliferation [83]. Furthermore, miR-34 has been discovered to attenuate the canonical Wnt-signalling via corporation with p53 in A549 and MCF-7 carcinoma cell lines. Knockdown and/or deletion of functional miR-34a gene resulted in the increased expression of WNT1, LRP6 and β-catenin mRNA in these cells [84]. The significance of the above findings is that β-catenin deregulation is a major hallmark of cancer. These results all suggest that miR-34, miR-320, miR-200 and Let-7 could be exploited for the development of therapeutic agents with specific focus on targeting the canonical Wnt-signalling pathway in cancer.

### Wnt/miRNA network in the regulation of cancer stem cells

**Fig. 2:**
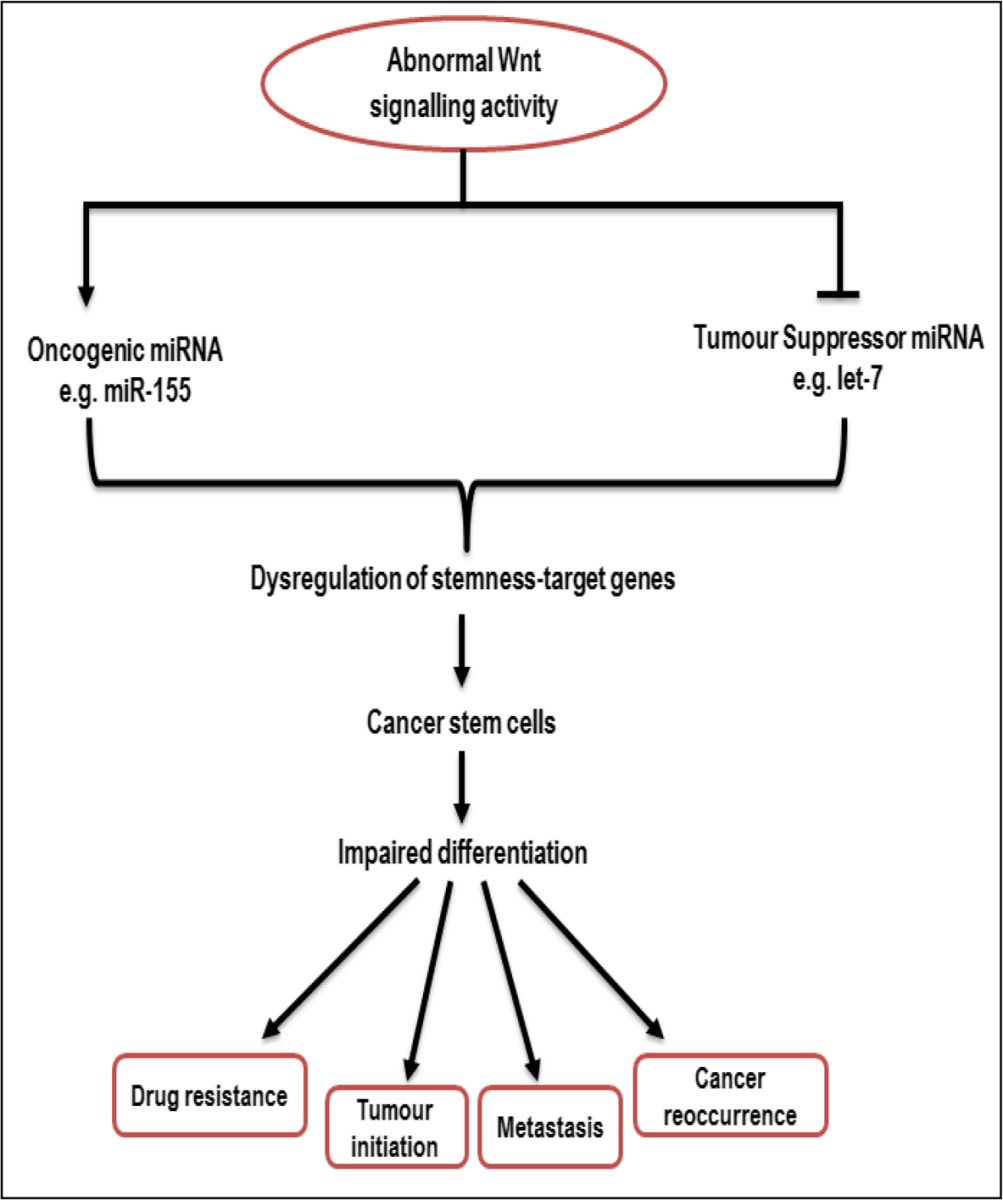
Importance of Wnt/miRNA network in the regulation of cancer stem-like cells. The Wnt-signalling (canonical & non-canonical) pathway governs the activity of some stem cell-specific miRNAs which plays crucial roles in tumour initiation and development. Aberrant Wnt-signalling could activate oncogenic miRNA expression which in-turn enhances the self-renewal potential of CSCs with subsequent expansion of the CSC pool which gives rise to cancer cells that possess self-renewing ability, resistance to drug therapy and ability to instigate new tumour growth

The controversial cancer stem cell (CSC) theory is based on the phenomenon that cancer cells may be derived from a rare population of cells possessing stem cell properties [85–87]. Experimental evidence from multiple studies suggests that CSCs possess a variety of biological properties similar to normal somatic stem cells such as the self-renewal capability, an integral program for tissue renewal and regeneration. The Wnt pathway plays crucial roles in the regulation of stem cell/progenitors, cell self-renewal and maintenance in a plethora of systems [10]. A major similarity between normal stem cell and CSCs is the fact that they both function via common signalling pathways such as Wnt & Notch pathways that aids in the maintenance of proliferation of stem cells [86]. In contrast, CSCs are distinctive due to the possession of several pro-cancer characteristics such as chemo-resistance and tumourigenic and metastatic activities that are not exhibited by their normal stem cell counterparts (Fig. 2).

Recent studies have established the importance and significant roles played by the canonical Wnt-signalling in stem cell self-renewal activities in various tissues and cell types [88]. For instance, Wnt3a was shown to foster the formation of embryonic stem cell (ESC)-like colonies [89]. Canonical Wnt pathway activation was shown to augment alkaline phosphatase and Cbfa1/Runx2 expression thereby facilitating mesenchymal stem cells (MSCs) osteogenic differentiation [90, 91]. Canonical Wnt-signalling is also crucial for differentiation and maintenance of the intestine as several scientific evidence have identified the expression of Wnt receptors and ligands such as LRP5, SFRP5, Wnt3/6, Fzd4/6/7 in epithelial cells located at the intestinal crypt base [88, 92].

The role of miRNA in the regulation of stemness of CSCs is another aspect of miRNA activity currently under investigation. Reports suggest that miR-193a expression inhibits tumourigenicity and invasiveness by directly targeting KRAS and plasminogen activator urokinase (PLAU), respectively, with these two factors highly expressed in human colon adenocarcinomas [93]. Also, miR-451 appears to be another regulator of CSC properties such as drug resistance, self-renewal and tumourigenicity. Experiments carried out in spheroid cell cultures, showed reduced expression of miR-451, triggering the up-regulation of macrophage migration inhibitory factor (MIF) and COX-2 which are involved in deregulation of canonical Wnt pathway in CSCs [94, 95]. Another miRNA, miR-34a inhibits Notch signalling by directly targeting Notch receptors [96], resulting in an impaired Notch signalling generation of daughter cells (non-CSCs), whereas low miR-34a levels enhances Notch signalling and in turn promotes maintenance of CSCs. Members of the miR-34 family of miRNAs which have also been implicated as direct targets of p53, act as tumour suppressors by governing reprogramming through the suppression of pluripotency genes including Sox2, Nanog and N-myc [97, 98]. Additionally, the let-7 family, also a negative regulator of β-catenin, is another crucial modulator of ESC differentiation [99] (Fig. 2).

### Cancer-related miRNA modulation of Wnt-signalling cascade

As critical biological modulators, it is no more news that miRNAs acts to suppress or facilitate cancer and tumour development by interacting with targets of the Wnt-signalling pathway. A summarized description showing the alterations of miRNAs/Wnt/β-catenin signalling in cancer has been provided (Table 1). A more detailed explanation on the Wnt/miRNA network in carcinogenesis has been provided in recent studies [100, 101].

**Table 1:** Alterations of miRNAs/Wnt/β-catenin signalling in cancer

Cancer type	miRNA	Wnt-signalling target(s)	Inhibits/activates carcinogenesis	References
Colorectal	miR-224	cdc42	Inhibits	[100]
Colorectal	miR-574-5p	Qki6/7, p27, β-catenin	Activates	[214]
Colorectal	miR-7,miR-34	Ying Yang	Activates	[215, 216]
Colorectal	miR-29b	BCL9	Inhibits	[217]
Liver	miR-155, miR-106b	*APC*	Activates	[218, 219]
Liver	miR-148b, miR-122	Wnt1	Inhibits	[220, 221]
Liver	miR-139	TCF4	Inhibits	[222]
Breast	miR-31, miR145	RhoA, β-catenin, c-myc	Inhibits	[223, 224]
Breast	miR-142	*APC*	Activates	[164]
Prostate	miR320	β-catenin	Inhibits	[225]
Lung	miR-191	Wnt1	Activates	[226]
Thyroid	miR-146b-5p	ZNRF3	Activates	[227]
Ovarian	miR-181a	VNGL1	Activates	[228]
Oesophageal	miR-141	SOX17	Activates	[229]

### Bioinformatics approaches to study Wnt pathway-regulated miRNAs and their targets

Following the initial discovery of the association of miRNA with cancer over more than a decade ago, technological advances that have produced multiple high-throughput bioinformatics methods designed for profiling miRNA expression and identification of miRNA targets involved in a plethora of pathways that regulate normal physiology and various diseases have being established [53]. Compared with other nucleic acids, miRNA analysis tends to be intricate due to several factors ranging from their short length, ability to discriminate between primary and mature forms and highly conserved sequences within family members. Notwithstanding, the evolution of Next-generation sequencing (NGS) platforms which allows for the simultaneous discovery of new miRNAs and confirmation of known miRNAs, overcome the limitations presented by microarrays and other traditional methods used for miRNA profiling [102, 103]. NGS effectively reduces the burden of genome sequencing by enabling the identification of characteristic expression level and splicing variants within the transcriptome [104, 105], characterization of DNA-protein interaction [106, 107] and understanding the role epigenetics plays in normal and diseased state [108, 109].

The challenging enormity of tumour heterogeneity, both in the primary tumour and metastasis has become explicit. Although, individual interactions between components of the canonical Wnt pathway and miRNAs in normal and cancer state have already been established [59, 71, 110, 111], we have simply just began to comprehend the role of miRNAs, not only in normal human development but also in tumour progression mediated by the canonical Wnt-signalling and vice-versa especially due to the fact that multiple components are involved in this complex network. Therefore, more efforts are required to identify the mechanism that controls miRNA/canonical Wnt-signalling network. As a result, bioinformatics strategies that provide a comprehensive, genome-wide identification of Wnt/β-catenin-regulated miRNAs and their associated target genes as well as other transcription factors that cross-talk with the pathway must be employed. The bioinformatics strategy adopted to execute this approach involves the identification of Wnt/β-catenin-regulated miRNAs or vice-versa and the identification of down-stream target mRNAs of the Wnt/β-catenin-regulated miRNAs undertaken via gene sequence complementarity. At the moment, numerous pilot projects comprising of cancer genomes have being undertaken using NGS in clinical research, mainly with the aim of identifying oncogenic mutations that can be exploited by mutation-specific drugs which would be useful for personalized medicine [112].

**Fig. 3:**
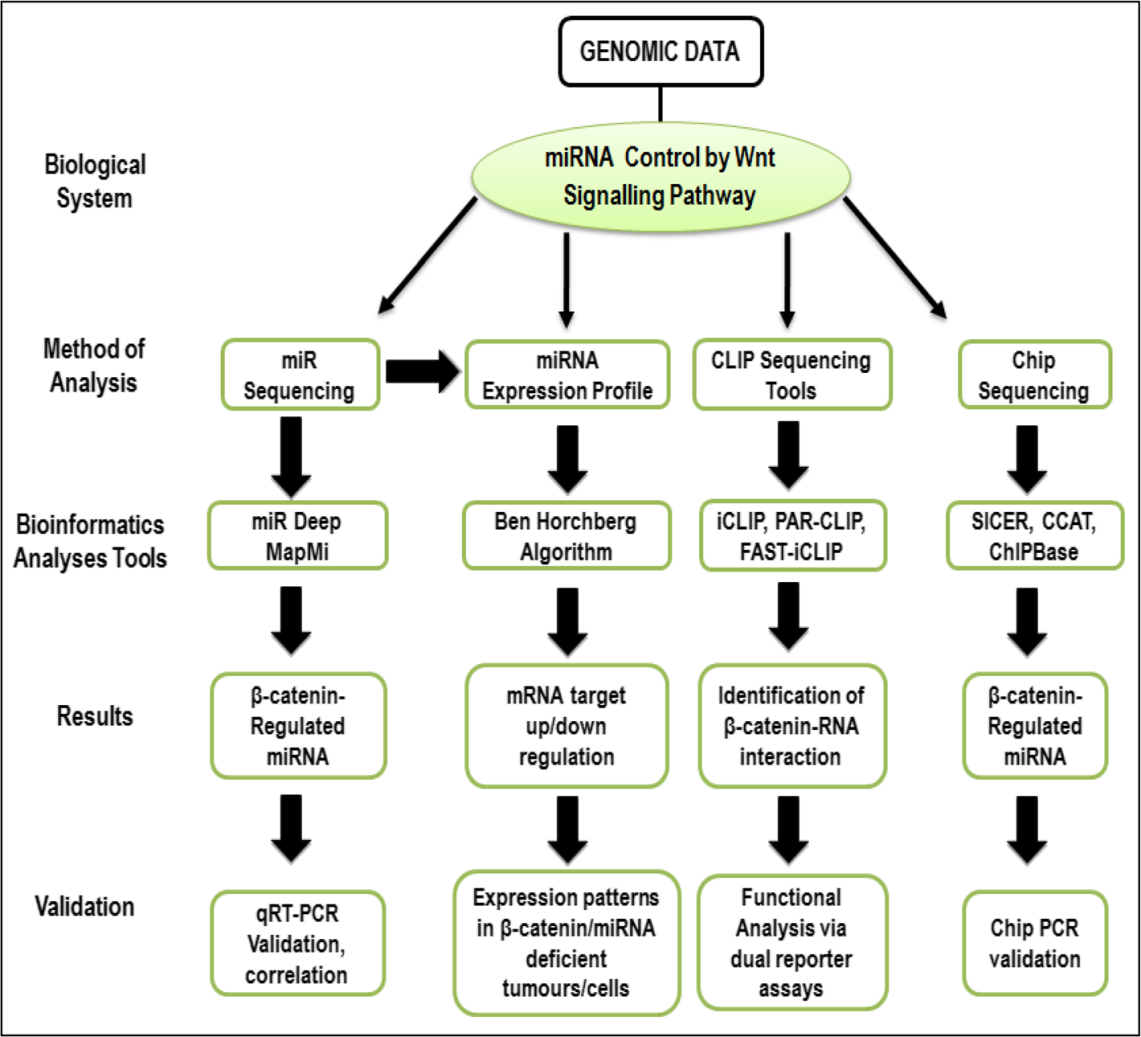
Bioinformatics pathway for the characterization of Wnt-signalling regulated miRNAs and their targets. Summary of a proposed stepwise bioinformatics approach for the characterization of Wnt-regulated miRNAs. Due to the fact that canonical Wnt pathway is driven by several components; key components such as β-catenin can be utilized to identify potential miRNA regulators. Programmes and tools such as Benjamin-Hochberg [210], iCLIP [211], miRDeep [212] have all been used to perform bioinformatics analysis for different experiments. Image adapted from [213]. Note: β-catenin is a major transducer in the canonical Wnt-signalling pathway hence; the proposed stepwise bioinformatics approach can be applied to the study of network of miRNA and other key components of either the canonical or non-canonical signalling pathway

In the following section we will briefly describe the different bioinformatics approaches that have been utilized in recent advances to identify and characterize Wnt/β-catenin-regulated miRNAs and their associated targets as well as the experimental strategies equally used for the validation of bioinformatics data (Fig. 3).

### ChIP-sequencing

**Fig. 4:**
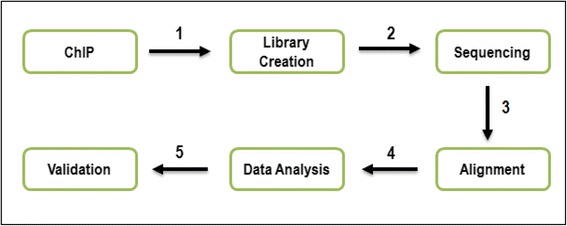
Flow chart illustrating the steps involved in the ChIP-Seq procedure

Recent advances in next-generation DNA sequencing combined with chromatin immuno-precipitation (ChIP-Seq) have provided methods ideal for identification of transcription factor binding sites (TFBSs) with exceptional sensitivity as well as predicting mRNA targets of miRNA [113]. ChIP has a reputation for being the gold standard technique for the identification of a target gene of various transcription factors [114, 115]. Technological advancement in ChIP studies allows investigators the luxury to utilize ChIP assay for the recognition and characterization of the entire binding sites for a particular transcription factor (Fig. 4). For instance, using the ChIP-Seq approaches, recent studies have identified 1250 overlapping putative target genes co-regulated by both TCF4 and STAT3 in gliomas [116]. TCF4, a member of the Tcf/Lef family and ubiquitously expressed in the colon epithelium, forms a complex with β-catenin with subsequent binding to promoter regions of specific genes that trigger processes crucial for normal development and in abnormal conditions, drive tumour progression [117, 118]. In recent times, miRNAs have been identified as one of the factors that possess the ability to modulate biological networks, including Wnt/β-catenin/TCF4 signalling. For instance, using IEC-6 cell nuclear extracts, putative binding sequences within the miR-30e promoter region were discovered, via ChIP assay, to actively bind beta-catenin/TCF4 with experiments suggesting miR-30e as a potential downstream target for the β-catenin/TCF4-mediated intestinal cell differentiation [119]. Further characterization using miRBase Sequence Database identified the conserved sequence of 5′-*UGUAAACAUCCUUGACUGGAAG*-3′ in mature miR-30e sequences derived from human, mouse, and rat. Additionally, the binding properties of β-catenin/TCF4 with miR-30e were validated and confirmed by performing EMSA and super-shift assay.

Similarly, ChIP experiments carried out in mESCs and P19 cells showed that Tcf3 binds directly to the miR-302 promoter regions to repress the transcription of the miR-302 gene [120], thus suggesting miR-302 gene is transcriptionally regulated via the Wnt/β-catenin pathway. Tcf3, a member of the Tcf/Lef family comprised of Tcf1, Tcf3, Tcf4 and Lef1 transcription factors mainly acts as a transcriptional repressor of the canonical Wnt-signalling by competing for β-catenin binding sites [121].

As previous studies have implicated mutations of *APC*, *CTNNB1* and *TCF4* genes and other components of the Wnt-signalling pathway as key oncogenic drivers in different forms of cancers [122, 123], applications of ChIP-Seq would be beneficial in providing a genome-wide binding profile of components of this pathway in other to identify potential miRNA genes that may be co-regulated by the Wnt-signalling pathway and exploited for therapeutic purposes [124]. Importantly, in the field of epigenetics, utilization of ChIP-Seq can also provide a platform with which epigenetic profiles through the study of primary cells and tissues can also be generated via identification of genome-wide profiles of DNA methylation, nucleosome positioning and histone modifications. This would provide a better understanding to the epigenetic machinery and alterations in the epigenetic landscape that occur in cancer compared with normal cells, the roles of these changes in cancer initiation, progression, metastasis and the potential use of this awareness in designing more effective therapeutic treatment strategies [125].

There have been several recent papers detailing ChIP-Seq limitations, for examples difficulty in transferring and maintaining data due to the large file sizes [126], and identifying regions enriched in the sample through the use of peak callers [127] that appears to be due to the presence of three types of regions namely, sharp, broad, and mixed. These shortcomings, can however be abolished by the development of more user-friendly software analysis tools in the near future.

### miRNA expression profiling

The comprehensive and efficient study of miRNA expression profiling has been made convenient via the evolution of next generation sequencing technologies. Our understanding of the regulatory roles of miRNAs in gene expression, and association with diseases triggering multiple changes in gene expression levels facilitating cancer and tumour progression has been improved upon by advances in miRNA expression profiling research [128], which could also be derived from miRNA seq (Fig. 3). Although high-throughput platforms such as microarray hybridization and real-time reverse transcription PCR have shown to be authentic tools and are still applicable in current research, next generation sequencing (NGS) technology has established itself as the most preferred and efficient platform for miRNA expression study as it possesses the ability to identify novel miRNAs that are beyond the capabilities of traditional methods [129].

Identification of Wnt-regulated miRNAs through expression profiling methods can be achieved by enforced expression of the Wnt ligand which consequently results to the subsequent activation of both or either canonical or non-canonical Wnt-signalling pathways depending on the type of Wnt ligand. Specifically, expression levels of individual components that are crucial to the progression of the cascade such as APC, β-catenin and Tcf4 transcription factors can be matched with different miRNAs with the aim of observing patterns of miRNAs regulated by the Wnt pathway. For instance, characterization of non-coding transcriptomes of tissues derived from six normal pancreas and pancreatic cancer (PDAC) tissues using high-throughput NGS-based technology algorithms such as small RNA-sequencing (sRNA-Seq) and Massive Analysis of cDNA Ends (MACE) identified miR-802 as a negative regulator of TCF4 [130], which upon binding to β-catenin triggers the expression of Wnt target genes. Interestingly, using omiRas [131], *TCF4* was observed to contain three mir-802 binding sites in its 3′ UTR with additional validation experiments revealing a significant correlation with Zeb1 and miR-21. Several studies have also confirmed Zeb1 as a key Wnt target [132, 133] as well as identification of miR-802 as a potential tumour suppressor via NGS techniques [134]. Some other popular NGS miRNA databases that can be beneficial in carrying out miRNA expression profiling for identification of Wnt target genes includes, but not restricted to: deepBase [135], miRGen 2.0 [136], miRBase [137], miRExpress [138] and CLIPseqtools [139].

Notwithstanding, traditional platforms for the execution of miRNA expression profiling have also been useful in identifying miRNA targets of the Wnt-signalling pathway and vice-versa. In colorectal cancer, global miRNA expression profiling carried out on 13 cancer and adjacent normal samples showed a significant down-regulation of 61 and up-regulation of 42 miRNAs with enrichment in pathways promoting tumour progression such as the Wnt pathway in addition to TGF-β and MAPK pathways [140]. Also, following differential miRNA expression profiling of HepG2 under varying conditions and validations performed by RT-PCR, 9 miRNAs, including miR-34a was observed to be differentially expressed following AFB1 treatment [141]. Aflatoxin-B1 (AFB1) is a hepato-carcinogenic mycotoxin that induces hepatocellular carcinoma [142–144]. Meanwhile, separate studies have implicated miR-34a as a tumour suppressor and negative regulator of the Wnt-signalling pathway [59, 145, 146].

A major limitation/complication of miRNA expression profiling includes the recent discovery of isomiRNAs that tend to display sequence variations by shortening or lengthening of the 3’ end [147, 148]. Unfortunately, recent studies have identified over 3300 miRNA variants with the most abundant miRNA sequence different from the miRBase sequence [149]. Additionally, miRNA end heterogeneity can influence the accuracy and consistency of quantifying miRNA expression levels. Due to the heavy reliance of qPCR and microarrays on the accuracy and availability of miRBase sequences for probe and primer design, mutations may contribute to difficulty in miRNA detection [148].

### CLIP/miRNA sequencing

Crosslinking and immunoprecipitation sequencing (CLIP-Seq) offers the luxury of analysing RNA/protein interaction. This approach is similar to RIP-Seq, however, stabilization of protein-RNA complexes are achieved by ultra-violet (UV) crosslinking. UV crosslinking is used as an ideal crosslinking agent in RNA studies due to the inability for UV crosslinks to form between proteins. miRNAs are subsequently reverse transcribed to cDNA and analysed with sequencing (RNA-Seq) for mapping of miRNA binding sites on their target mRNAs with high confidence [150]. These sets of sequenced miRNA target and pathway genes are subsequently screened to identify its associated biological pathways for a comprehensive understanding of their biological function.

CLIP-Seq and target prediction studies effectively identify individual mRNAs regulated by multiple miRNAs, therefore proving that the transcriptional regulation of a single gene may be dependent on the combined effect of multiple miRNAs. Next-generation sequencing of RNA (RNA-Seq) allows for the generation of tissue-specific gene expression profile data which can be useful in developing a novel pathway analysis methodology for the prediction of miRNA function. Some web applications used for the identification of miRNA-regulated pathways in a tissue specific manner include miTALOS v2 [150], ToppMir [151] and miRGator [152].

Although experimental studies focused specifically on the identification of miRNAs that regulate the Wnt-signalling pathway are scarce, pathway analysis using CLIP-Seq of the miR-200 cluster family (miR-200b/c/miR-429 and miR-200a/miR-141) which has been established to be involved in cell migration and EMT, was able to identify a connection between miR-200b/c/miR-429 and the Wnt-signalling pathway in hepatocellular carcinoma (HCC) and liver fibrosis tissue samples [150]. Additionally, miR-199a/b-3p was also implicated to be involved in EMT, cell migration and metastasis through cytoskeletal re-organization. Interestingly, this was in agreement with previous studies describing the involvement of miR-199 in EMT [153, 154].

A major limitation of CLIP-Seq however is the inability to reduce background noise/signals due to the nature of high-throughput sequencing. For instance, Matthew et al. [155] reported the difficulties in eliminating cross-linked background containing T > C conversions by bioinformatics analysis. Another bottleneck is the low RNA output efficiency inevitably due to the loss of RNA content and low cross-linking efficiency during the experimental process [156].

### Validation and follow-up experiments

Following the elucidation of Wnt-mediated regulation of miRNAs or vice versa, potential candidate targets are confirmed and validated by additional experimental analyses that interrogate the pathological and physiological implications of the discovered regulations. These validations can be performed via two conventional strategies: an approach involving the exposure of a tumour tissue/sample to genetically altered oncogenic candidates, or an approach which involves the systematic manipulation of oncogenic candidates into becoming wild-type tissues [157]. The latter can be further modified by introducing the oncogenic or tumour suppressor candidate into a different mouse model (immunocompetent of immuno-compromised) for the purpose of gain of function and loss of function studies on a potential miRNA target.

### CRISPR/Cas9 System

Gain-and-loss-of-function studies are one of the most efficient approaches employed to validate the oncogenic/tumour suppressor potential of a target gene following NGS methodologies. However, in comparison to the relative potency of numerous overexpression strategies, the methodologies developed for miRNA downregulation appear to be less robust [158, 159]. More recently, the CRISPR (clustered regularly interspaced short palindromic repeats) became a well-recognized genome editing tool, referred to as CRISPR-associated endonuclease (Cas9) system [160]. CRISPR consists of short palindromic repeat sequences interspacing with spacers adjacent to associated endonucleases, such as Cas9. Considering the difficulties in contemporary methodologies in miRNA silencing versus the versatility and flexibility of CRISPR/cas9 system in gene editing, CRISPR/cas9 should easily be an unorthodox strategy in the regulation of miRNA expression [159].

For instance, using HT29 and HCT116 CRC cells transfected CRISPR/cas9 vectors were able to reduce the expression levels of mature miR-200c as well as miR-141 and miR-17 by 96 % [159], with recent evidences implicating miR200 as a key modulator of canonical Wnt-signalling [70, 161, 162]. This supports the hypothesis that CRISPR/cas9 system can be a suitable tool for miRNA loss-of-function validation studies following bioinformatics analysis. Furthermore, following transcriptional profiling of MV4-11 B-myelomonocytic leukaemia cell lines using RNA sequencing, CRISPR/cas9 technologies enabled the validation of miR-150 as a bonafide oncogenic promoter of leukemic cell proliferation and growth through targeting of p53 [163]. This is also consistent with previous studies suggesting that the activation of canonical Wnt-signalling pathway and miR-150 in human breast cancer stem cells (BCSCs) is modulated by miR-142 [164]. Similarly, microRNA profiling analyses in conjunction with CRISPR/Cas9 systems have been utilized to validate candidate novel transcription factors including miR-199 which was revealed as an oncogenic activator involved in Pancreatic ductal adenocarcinoma (PDAC) pathogenesis [165]. Here in, analysis of miR-199 functional significance in pancreatic cancer further showed induction in migration, invasion and proliferation triggered by miR-199 inhibition of FOXA2. This is also in agreement with previous study suggesting miR-199 targets several key differentiation and cell proliferation regulatory factors governing the Wnt-signalling pathway, such as fzd4 [166]. All the above mentioned examples are a testament to the ever increasing relevance of the CRISPR/Cas9 system in the validation of specific miRNAs involved in, but not restricted to the Wnt-signalling pathway in cancer and tumour progression.

Despite the outstanding potential of CRISPR/Cas9 in transcription regulation, genome editing and gene therapy, some important issues, such as off-target mutations [167], PAM dependence [168] and gRNA production [169], all provides a bottleneck to the efficiency of system. Several miRNAs reside in the introns of their pre-mRNA host genes and share common regulatory elements, primary transcripts, resulting to similar expression profile patterns. This makes it difficult to distinguish the functional effects arising from both the miRNA silencing and host gene silencing. Nevertheless, alteration of the first 20 nucleotide sequences of the gRNA to hybridize to target DNA sequence can be utilized to distinguish the functional effects of miRNA genes transcribed from their own promoters [170]. Although DNA and RNA injection-based techniques such as inoculation of CRISPR components as RNA and plasmids expressing gRNA and Cas9 is possible, more attention should still be focussed on development of novel robust delivery methods for CRISPR/Cas9 system [171].

### Genetically engineered mouse models (GEMMs)

The use of genetically engineered mouse models (GEMMs) can also provide an avenue for the validation of miRNAs involved in the regulation of Wnt-signalling pathway following bioinformatics analysis. Although there exist some differences between humans and mice, new models possess the ability to accurately mimic erratic human malignancies and tumour development, thus enabling efficient tracking of both primary and metastatic tumour progression from initial stages than hitherto possible [172]. These mouse models have particularly improved our knowledge of cancer initiation, metastasis, and invasion, tumour angiogenesis as well as the importance of the myriad of molecular networks observed in human cancers. The study of loss-of gene function is also applicable in mouse models by performing mouse conditional gene mutation [173], mouse gene knock-outs (KO) [174, 175], mouse single cell knock-outs [176] and mouse models for RNA interference [177, 178]. Similarly, gain of gene function studies in mouse models have been successfully performed [172], however for the purpose of this review we will specifically focus on mouse models for RNA interference studies.

To elucidate the importance of miR-184 in modulating Wnt-signalling in the retina, delivery of formulated nanoparticle-derived miR-184 in the retina of oxygen-induced retinopathy (OIR) mice significantly inhibited Wnt-signalling [179]. OIR is an established model for the study of vascular pathology in the retina [180, 181]. Additionally, a more recent study utilized the generation of an endothelial-specific miR-17 ~ 92 cluster knock-out mice by crossing mice possessing a floxed miR-17 ∼ 92 allele transgenic mice expressing Cre-recombinase under the control of a tamoxifen-inducible CDH5 (VE-cadherin) promoter [182, 183]. This model enabled the identification of Fzd4 and LRP6 receptors as functionally pertinent miR-19 target genes with further studies on 17 ∼ 92 KO mice suggesting miR-17 ~ 92 cluster antagonizes the canonical Wnt-signalling cascade [183]. In separate studies, the existence of a let-7/Lin28/Wnt-β-catenin signalling network *in-vivo* was confirmed using premalignant mammary tissue of MMTV-Wnt-1transgenic mice [184]. Again, the above mentioned examples amongst numerous others have highlights the relevance of GEMMs as an authentic experimental approach for the validation of miRNAs as targets of Wnt-β-catenin signalling pathway following bioinformatics data analysis.

### Intestinal organoid culture

The evolution of tissue engineering have given rise to organoid culture which is a novel and effective tissue stem cell derived three dimensional (3D) model and a useful tool predominantly for functional study [185, 186]. Basically, glandular organ 3D culture is categorized into those derived from either a combination of both mesenchymal and epithelial components from stomach, colon, liver, lung and small intestines supplemented by exogenous growth factors [187–189] or specifically from epithelial cells of gastrointestinal tissue origin [190–192]. Potential applications of organoid models include mainly functional validation studies such as validation of putative oncogenic or tumour suppressor genes and cancer therapeutic validation studies [189, 193, 194]. It is therefore not surprising that this model have already been exploited to validate the Wnt-derived oncogenic and tumour suppressor properties of miRNAs.

Contextual modelling in *APC*-deleted colon-derived organoids overexpressing either miR-483 or Igf2 showed that enforced miR-483 expression promotes high-grade dysplasia [193]. Although experimental evidences have implicated miR-142 as a tumour suppressor in breast cancer cells *in vivo* [195, 196], others have suggested that miR-142 maintains breast cancer stem cells (BCSCs) by activating the canonical Wnt-signalling pathway [164]. The ability of miR-142 to modulate organoid formation in BCSCs was further investigated with results suggesting miR-142-3p as an essential regulator of organoid formation in murine mammary CSCs [164]. Furthermore, in a separate study, primary mammary epithelial organoids were derived from axin2/conductin-lacZ mouse [197] in order to determine whether candidate miRs can control Wnt-signalling with results strongly implicating miR-1 as an inhibitor of Wnt-signalling pathway *in vivo* [198]. The relatively rigid ECM (Matrigel) could serve as a limiting factor by obstructing drug penetration, thereby hindering the effectiveness of organoids in drug screens via robust lentiviral or miRNA delivery systems. Although organoid protocols have been established for tissues derived from various organs [199], more efforts needs to be applied to the development of organoids culture from tissues whose niche factors remain poorly understood [200].

Apart from the above mentioned techniques and experimental designs utilized in the validation of the gene expression levels and numerous short sequences generated by NGS platforms, other validation techniques widely used include quantitative RT-PCR which is often used for the validation of differentially expressed genes discovered using microarray and RNA-Seq [201, 202]. Functional validation of novel miRNAs involved in Wnt-signalling pathway can also be performed via induced pluripotent stem cells (iPSC) technologies. Although not used specifically for the validation of miRNA/Wnt-signalling network in cancer, patient-derived iPSC approaches [203], have been employed in other studies for validation of NGS and other gene sequencing-based data [204, 205]. Additionally, guidelines for the validation of clinical data acquired from NGS are also available to safeguard the standard of clinical NGS experiments [206–208].

## Conclusions

**Fig. 5:**
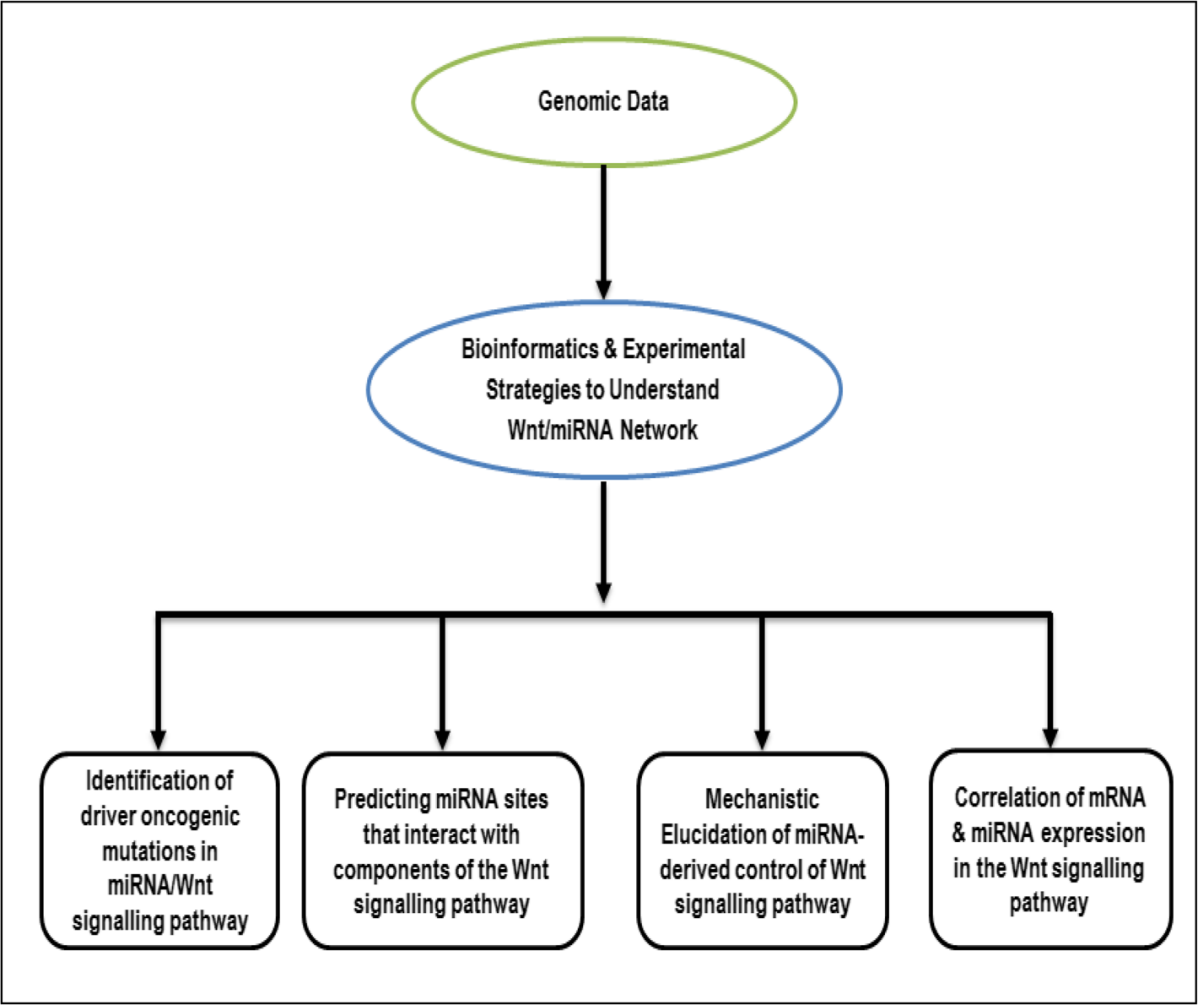
Schematic overview of benefits of bioinformatics and experimental approaches in understanding miRNA/Wnt-signalling network in cancer

Although the application of bioinformatics in the elucidation of the miRNAs-mediated regulation of Wnt-signalling is extremely beneficial (Fig. 5), particularly in the area of cancer research, the significant challenges that remain still signify the usefulness of experimental approaches for the analysis and validation of bioinformatics data. The misconception that a dichotomy exists between bioinformatics and experimental approaches in cancer research would certainly slow down the rate of progress in establishing the roles miRNA plays in the physiology and pathology of the human system. While bioinformatics strategies may be seen as improvements to experimental approaches such as the so-called ‘wet lab experiments’, we cannot deny the fact that experimental approaches are still relevant in modern medicine, especially in the area of experimental validation. As already noted in this review, the NGS system still possesses significant challenges; hence in order to circumvent the bottleneck of data storage as well as complex data analysis, the continual development of methodologies/algorithms for data analysis and integration is necessary. Given the ever increasing passion for the identification of proteomic and genomic biomarkers to enhance cancer detection at the early stage, utilization of both bioinformatics and experimental strategies for miRNA target identification, functional target validation and their specificity for particular tissues must be employed. Further investigations of the miRNA/Wnt-signalling network, in addition to the crosstalk between miRNAs and other signalling networks implicated in cancer development must also be performed in order to boost productive application to improve human health.

## Abbreviations

APC: Adenomatous polyposis coli
CAF: Cancer associated fibroblasts
CaMK: Calcium/calmodulin-dependent kinase
ChIP-Seq: Chromatin IP Sequencing
CK1: Casein kinase1
CLIP-Seq: Crosslinking and immunoprecipitation sequencing
CRC: Colorectal cancer
CRISPR: Clustered regularly interspaced short palindromic repeats
CRISPR: Clustered regularly interspaced short palindromic repeats
CSC: Cancer stem cell
EMT: Epithelial-mesenchymal transition
ESC: Embryonic stem cells
GSK-3β: Glycogen synthase kinase 3β
HCC: Hepatocellular carcinoma
iPSC: Induced pluripotent stem cells
MACE: Massive Analysis of cDNA Ends
miRNAs: microRNAs
NFAT: Nuclear factor of activated T-cells
NGS: Next generation sequencing
NSCLC: Non -small cell lung cancer
PKC: Protein kinase C
PLAU: Plasminogen activator urokinase
sRNA-Seq: Small RNA-sequencing
UTR: Un-translated region
β-TRCP: β-transducin repeat-containing protein.
